# The Use of Proton Pump Inhibitors May Increase Symptoms of Muscle Function Loss in Patients with Chronic Illnesses

**DOI:** 10.3390/ijms21010323

**Published:** 2020-01-03

**Authors:** Paulien Vinke, Evertine Wesselink, Wout van Orten-Luiten, Klaske van Norren

**Affiliations:** 1 Nutritional Biology, Division of Human Nutrition and Health, Wageningen University, Stippeneng 4, 6708 WE Wageningen, The Netherlands; paulien.vinke@wur.nl (P.V.); wout.vanorten-luiten@wur.nl (W.v.O.-L.); 2 Heart Center Leipzig at University of Leipzig, Department of Internal Medicine/Cardiology, Strümpellstraße 39, 04289 Leipzig, Germany; 3 Nutrition and Disease, Division of Human Nutrition and Health, Wageningen University, Stippeneng 4, 6708 WE Wageningen, The Netherlands; vera.wesselink@wur.nl; 4 Department of Geriatric Medicine, Gelderse Vallei Hospital, Willy Brandtlaan 10, 6716RP Ede, The Netherlands

**Keywords:** proton pump inhibitors, cachexia, inflammation, sarcopenic obesity, cancer, COPD, heart failure, magnesium, vitamin D, microbiota

## Abstract

Long-term use of proton pump inhibitors (PPIs) is common in patients with muscle wasting-related chronic diseases. We explored the hypothesis that the use of PPIs may contribute to a reduction in muscle mass and function in these patients. Literature indicates that a PPI-induced reduction in acidity of the gastrointestinal tract can decrease the absorption of, amongst others, magnesium. Low levels of magnesium are associated with impaired muscle function. This unwanted side-effect of PPIs on muscle function has been described in different disease backgrounds. Furthermore, magnesium is necessary for activation of vitamin D. Low vitamin D and magnesium levels together can lead to increased inflammation involved in muscle wasting. In addition, PPI use has been described to alter the microbiota’s composition in the gut, which might lead to increased inflammation. However, PPIs are often provided together with nonsteroidal anti-inflammatory drugs (NSAIDs), which are anti-inflammatory. In the presence of obesity, additional mechanisms could further contribute to muscle alterations. In conclusion, use of PPIs has been reported to contribute to muscle function loss. Whether this will add to the risk factor for development of muscle function loss in patients with chronic disease needs further investigation.

## 1. Introduction

### 1.1. Cachexia in Chronic Illness

There are several definitions of cachexia, but in general, the term includes weight loss, low body mass index, fatigue, imbalance between anabolic and catabolic metabolic pathways, and upregulation of biomarkers of systemic inflammation [1,2,3,4,5]. In malnutrition, weight and muscle loss are reversible when adequate amounts of energy and protein are provided. However, in cachexia this is not the case [6]. A consensus definition has been proposed by Evans and colleagues: “Cachexia is defined as a loss of lean tissue mass, involving weight loss greater than 5% of body weight in 12 months or less in the presence of chronic illness or as a body mass index (BMI) lower than 20 kg/m^2^. In addition, three of the following five criteria are also required: decreased muscle strength, fatigue, anorexia, low fat-free mass index, an increase of inflammation markers, anemia, or low serum albumin” [2,7]. Cachexia is a serious complication that frequently occurs at advanced stage of many chronic illnesses, including cancer, chronic obstructive pulmonary disease (COPD), and cardiovascular disease. Cachexia affects the quality of life and survival of the patients. The prevalence of cachexia ranges between 15% and 90% and is dependent on the underlying disease [6,8]. Mortality rates of patients are also disease-dependent and range from 15% up to 80% [6]. Muscle mass and muscle function are to a large extent, related. It is only recently that it has become apparent that preservation of only muscle mass without improving muscle function has no effect on quality of life and mortality rates in cancer patients [9]. Apparently, loss of muscle function is contributing to a higher extent to morbidity and mortality than loss of muscle mass. Moreover, for age-related muscle loss, it has been described that loss of muscle function, specifically gait speed, correlates to morbidity and mortality [10]. Because most chronically ill patients are older people, this further strengthens the importance of muscle function in the chronically ill patient population. 

### 1.2. Pathophysiology of Cachexia

Cachexia is a complex and multifactorial disease [11,12]. Chronic upregulation of inflammatory mediators, such as interleukin-1 (IL-1), IL-6, tumor necrosis factor-α (TNF-α), interferon-γ (IFN-γ), and prostaglandin E2 (PGE2) [12,13,14] are an important part of its pathophysiology [6]. These inflammatory mediators, for example, affect appetite-regulating hormones in the hypothalamus, leading to lower food intake [15,16]. Another important contributor to cachexia is the imbalance between anabolic and catabolic metabolic pathways. This is also partly due to inflammatory processes, but many other mechanisms play important roles as well. As a consequence of the metabolic imbalance, muscle breakdown is increased and muscle synthesis is decreased, leading to lower muscle mass, lower muscle strength, and a sub-optimal body composition [6]. Moreover, muscle function further decreases, because muscle energy metabolism, including mitochondrial functioning, is impaired [17,18,19]. In some cachectic patients, the gut microbiome is altered as well. In cancer cachexia, gut permeability was found to be increased, whereas villi length, crypt dept, cecal content, and tissue weight were reported to be decreased. The inflammatory cytokine IL-6 was found to be a main driver behind these alterations in the gut [20].

### 1.3. Sarcopenic Obesity in Chronic Illness

Sarcopenia in the general sense refers to a condition of reduced muscle mass and functionality. Its underlying causes are diverse and include the process of normal aging. Sarcopenia is an important component of cachexia. The situation in which sarcopenia goes along with obesity is called sarcopenic obesity. This combination is also seen with cachexia and is in cancer independently associated with higher mortality and rate of complications in systemic and surgical cancer treatment [21,22,23,24]. The prevalence of sarcopenic obesity in advanced solid tumor patient populations is around 9% (range 2.3–14.6%). In this group, even one in four (24.7%, range 5.9–39.2%) of the patients with a body mass index ≥30 kg/m^2^ is also sarcopenic [21]. Excess visceral adipose tissue seems to be particularly risk increasing, whereas subcutaneous adipose tissues appears to be associated with a reduction in overall mortality risk [22]. This apparent paradox suggests that adipose tissue inflammation, which predominantly occurs in visceral adipose tissue, might play a key role. In cardiovascular diseases and heart failure, this obesity paradox is also frequently reported. A leading theory explains the paradox with the heterogeneity of the obese population, in which different phenotypes occur based on inflammatory characteristics and the relative amount of visceral fat. The three most extreme phenotypes of this hypothesis are formed by the metabolically healthy obesity (MHOB), describing an obese phenotype without metabolic syndrome, metabolically unhealthy obesity (MUO), and the metabolically obese normal weight (MONW) with normal BMI and obesity-related metabolic complications [25]. It seems that with cancer, being obese can be beneficial and reduce mortality risk, as long as the adipose tissue is predominantly subcutaneous, whereas being obese with predominantly visceral (inflammatory) adipose tissue can be harmful and contribute to sarcopenic obesity.

### 1.4. Adverse Effects of Long Term Use of PPIs

PPIs are among the most widely used medications worldwide, having high prescription rates, especially for the elderly [26,27,28]. Many patients who suffer from chronic diseases use proton pump inhibitors to reduce the risk of gastric ulceration and ulcer-related bleeding caused by chronic use of nonsteroidal anti-inflammatory drugs (NSAIDs) [29,30,31]. PPIs decrease the acidity in the stomach by inhibition of the H^+^/K^+^-ATPase enzyme in the parietal cells of the gastric glands. As a consequence, the pH of the proximal intestine is increased [29,32], which can affect the gut microbiome [33,34]. The use of PPIs has also been associated with a decreased absorption of vitamin B_12_, magnesium, calcium, iron, vitamin C, β-carotene, and zinc, which is at least in part a direct consequence of their reduced release from the food matrix and (or) effects on the absorption process [29,35,36,37,38] At the clinical level, PPI use has been reported as a variable of importance, after gait speed, oxidative stress, and depression, in the prediction of muscle mass and function loss in aging (sarcopenia) [39]. Moreover, it has been reported that long term use of PPIs can result in polymyositis, a type of chronic inflammation in the muscles [40]. Another possible, but rare side-effect of PPI use is rhabdomyolysis, a condition in which damaged muscle breaks down rapidly, and the breakdown product can effect kidney function [41,42]. To conclude, although generally considered safe, long-term use of PPIs could ultimately lead to deficiencies in several vitamins and minerals [36,37,43]. Furthermore, the changed pH can affect the gut microbiome [33,34], and finally, it can result in the presence of myopathy, which might affect muscle function and mass [39].

### 1.5. Aim and Scope

The adverse effects of long term PPI use can possibly impact the development of muscle mass and function loss [44] and influence the risk of developing cachexia in the presence or absence of obesity [20,45]. The aim of this paper is to elaborate on the possible effects of PPI use on muscle function and mass in individuals prone to loss of muscle mass and function, such as chronically ill patients. We focus on the possible effects of PPI use on magnesium levels and vitamin D metabolism, which may impact inflammatory processes in the body that are relevant in the development of muscle mass and function loss in chronic illnesses [46] and on alterations in the gut microbiota [12]. Next to that, we evaluate the impact of these deficiencies in a subtype of cachexia patients: the sarcopenic obese patients, because the presence of obesity might further enhance the impact of a reduction in these micronutrient levels. Additionally, we discuss the relation between PPI use, micronutrient status, and muscle function.

Proton pump inhibitors can influence the levels of certain micronutrients, such as magnesium, which in turn can influence vitamin D status. Changes in the levels of these micronutrients can enhance the development of chronic low-grade inflammation in chronic disease. 

## 2. Relation between PPI Use, Micronutrient Status and Muscle Function

### 2.1. PPI Use Is Associated with Low Magnesium Status

Magnesium functions as a second messenger molecule in many inflammatory processes [47] and is essential in a few hundred enzymatic reactions [47,48]. A normal range of serum magnesium levels is 0.7–1.07 mmol/L (or 1.7–2.6 mg/dL). Magnesium is mostly stored in the bone, muscles, and organs, but lower quantities can be found in the extracellular fluid [47,48]. Average magnesium ingestion from most diets is about 300 mg/day of which 100 mg/day is actually absorbed by the intestine [47,48]. Absorption occurs by a paracellular passive pathway and by active transcellular pathways via the receptors transient receptors potential melastatin 6 and 7 (TRPM6 and TRPM7) [47]. Epidemiological studies have shown that low levels of magnesium are associated with several chronic illnesses, such as diabetes and cardiovascular disease [47,48].

Hypomagnesemia (serum magnesium below 0.7 mmol/L) is a known side-effect of PPI use [29,35,36,37,38]. PPIs may decrease intestinal magnesium absorption by interfering with passive transport in the ileum and with active transport in the large intestine [48,49,50,51]. The activity of TRMP6 is regulated by intracellular magnesium along with pH, where a lower pH increases TRMP6 activity. It is hypothesized that PPI use could potentially decrease TRPM6 activity, resulting in decreased magnesium absorption [48,49]. The prevalence of hypomagnesemia in patients using PPI has been reported to range of 11–55% [52,53]. In two recent meta-analyses of nine observational studies with a total of 115,455 and 109,798 patients, pooled relative risks of hypomagnesemia in patients with PPI use were 1.78 (95% CI 1.08–2.92) [52] and 1.43 (95% CI 1.08–1.88) [54], respectively. It should be noted that data of six out of a total of 12 original studies were used in both meta-analyses [52,54]. In a study in hospitalized elderly using PPI, a hazard risk of 1.80 (95% CI 1.20–2.72) was observed [53]. Although results of observational studies showed an increased risk of hypomagnesemia in PPI users, the mechanisms by which PPIs induce hypomagnesemia are still being investigated.

### 2.2. Low Magnesium Levels Are Related to Decreased Muscle Function

Epidemiological data show that serum magnesium levels are independently correlated with muscle performance in older individuals [55]. In a cross-sectional study with adult women (18–79 years), a higher magnesium intake was associated with better indices of skeletal muscle mass and some but not all measures of muscle strength. The association was higher for younger age groups than for older age groups [56]. Recently, it was also shown that magnesium intake is associated with the prevalence of sarcopenia [57,58]. 

That magnesium supplementation can improve physical performance in elderly women in a weekly exercise program, was shown in a randomised controlled trial [59]. Magnesium is thought to help with muscle relaxation and so improve muscle function [60]. Magnesium plays a role in the muscle as a physiological antagonist of calcium, which is also responsible for muscle contraction. Muscle cramps and muscle weakness are indeed often seen in individuals with hypomagnesemia [48]. Systematic reviews have not shown any reduction in muscle cramps after magnesium supplementation in the general public [61], although it might be beneficial during pregnancy [62]. Because the studies were limited by a small patient population, more research is needed to study the effect of magnesium supplementation on muscle cramps in specific populations. 

Next to the effects on muscle function, low magnesium status can also stimulate muscle breakdown. In observational studies, magnesium intake was found to be inversely related to cachexia-associated inflammatory cytokines [63] such as CRP, TNFα, and IL-6, in a dose-dependent manner [64]. At present, more evidence is accumulating that low magnesium levels can contribute to a state of chronic low-grade inflammation in the body [47,64,65,66]. Because magnesium acts as a natural calcium antagonist in a number of processes, the inflammatory response is probably a result of changes in intracellular calcium concentrations by opening of calcium channels and activation of the *N*-methyl-d-aspartate (NMDA) receptor. Other possible mechanisms behind its effects on inflammation include activation of phagocytic cells; release of neurotransmitters, such as substance P; ROS production; and activation of NF-κB. Magnesium deficiency can also induce a systemic stress response through neuroendocrinological pathways [47,65]. 

### 2.3. Magnesium and Vitamin D Deficiency, Together, Can Lead to Increased Inflammation Affecting the Muscle

Next to direct effects of magnesium on inflammation, low magnesium levels may also indirectly increase levels of inflammatory cytokines by inhibition of the production of the active form of vitamin D, 1,25(OH)_2_D_3_, since magnesium plays a key role in vitamin D metabolism [67]. Vitamin D is derived from the production of vitamin D3 in the skin, via a non-enzymatic two-step process induced by UVB radiation and heat, or otherwise obtained from the diet [68]. In the blood, pre-vitamin D is transported by vitamin D binding protein (VDBP). The production of VDBP is magnesium-dependent; therefore, low magnesium levels may lead to lower levels of circulating VDBP, resulting in less transportation of vitamin D to the liver and kidney [68]. In the liver, vitamin D is converted by 25-hydroxylase to 25-hydroxy vitamin D3 (25(OH)D_3_). Finally, 25(OH)D_3_ is converted to its active form 1,25(OH)_2_D_3_ by 1-α hydroxylase in the kidney [68]. Both enzymes are magnesium-dependent [69]. So lower magnesium levels may lead to hampered production of the active form of vitamin D. 

Vitamin D plays a crucial role in many physiological functions, including bone and muscle metabolism [70]. Associations between low vitamin D levels and muscle metabolism disorders have been reported during aging and disease, leading to loss of muscle mass and function [44]. This effect is potentially mediated by the role of vitamin D in inflammation. The active form of vitamin D, 1,25(OH)_2_D_3_, also has anti-inflammatory effects [71,72]. Two main mechanisms by which 1,25(OH)D_3_ exerts anti-inflammatory responses have been suggested. First, 1,25(OH)_2_D_3_ may inhibit nuclear factor kappa B (NFκB) signalling [73]. NFκB is an important transcription factor involved in the regulation of inflammation. Activation results in the production of pro-inflammatory cytokines [74]. Second, 1,25(OH)_2_D_3_ may suppress p38 stress kinase signalling through the upregulation of mitogen-activated protein kinase phosphate 5, resulting in an inhibition of pro-inflammatory cytokine production, such as interleukin 6 (IL-6) [73]. Inflammatory cytokines, especially IL-6, are an important driver of muscle wasting in chronic disease that leads to cachexia. 

## 3. PPI Use and the Gut Microbiota in Relation with Cachexia

### 3.1. The Gut Microbiota Is Altered by PPI Use

Proton pump inhibitors elevate the pH of the stomach and upper gut, which enables more bacteria, including those with pathogenic properties, to survive gastric passage and enter the gut. A study confirmed this by showing that bacteria from the oral cavity are more abundant in the gut microbiomes of PPI users [34]. The authors also found that PPI use is associated with a decrease in bacterial richness and considerable changes in the gut microbiome, including significantly increased levels of the class Gammaproteobacteria, the family *Enterobacteriaceae* and the genus *Enterococcus*. Increased levels of these bacteria are associated with *Clostridium difficile* infections in both humans and animal models [34]. Several other studies also show that PPI use is associated with increased risk of enteric infections [75,76,77,78]. These shifts in composition of the microbiota may have immunological consequences, including an elevated pro-inflammatory status.

Interestingly, mice on a magnesium-deficient diet were found to develop a microbiota composition that is considered less favorable for health and an attenuated gut barrier function compared to mice on a magnesium sufficient diet [79]. This supports a link between low magnesium levels and a less healthy gut microbiota that affect inflammation and metabolic disorders [79]. 

**Figure 1:**
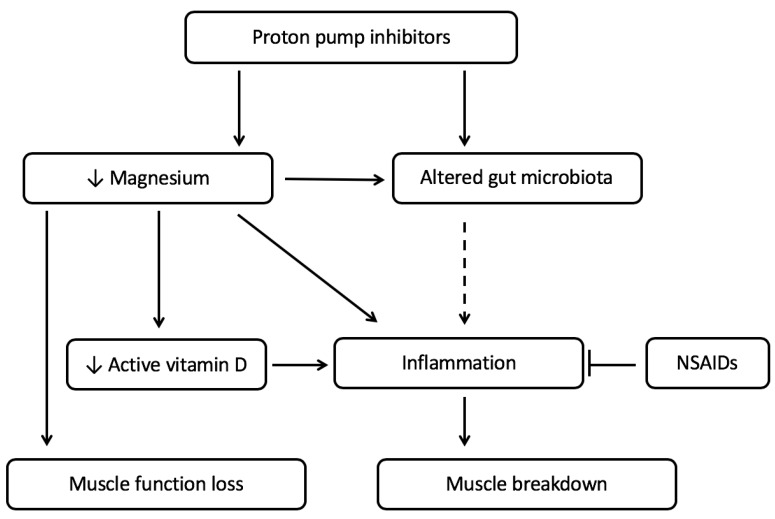
The proposed mechanism by which the use of proton pump inhibitors can lead to increased muscle function loss and increased muscle breakdown in cachexia-related chronic diseases. The use of proton pump inhibitors leads to an increase in chronic low-grade inflammation by altering the gut microbiota and decreasing magnesium and vitamin D levels. Lower magnesium levels lead to muscle function loss and increase inflammation directly and indirectly via vitamin D. The increase in inflammation leads to muscle breakdown. When PPIs are given together with NSAIDs, it is likely that the effect on inflammation is abandoned. The impact of PPI use on muscle function is likely not affected by the use of NSAIDs.

Moreover, a small human study suggests that consumption of inulin can improve blood magnesium concentrations in proton pump inhibitor-induced hypomagnesaemia. The explanation of the effect may lie in a combination of changed pH levels of the colon and a change in microbiota [80]. Interestingly, the study of Winther and colleagues showed [81] that a magnesium-deficient diet altered the gut microbiota of mice, and this led to depression-like behavior. The altered gut microbiota also correlated positively with IL-6 levels in the hippocampus, suggesting that inflammatory processes in the brain played a role [81]. As discussed before, similar inflammatory mediators have been shown to affect appetite-regulating hormones in the hypothalamus, leading to lower food intake [15,16]. What has to be taken into account, however, is that PPIs are often taken together with NSAIDs that will counteract this effect. In view of these findings, crosstalk between the PPI-induced processes leading to inflammation might occur when PPIs are provided in the absence of NSAIDs (Figure 1), while the effect on muscle function is more likely to occur both in the presence and absence of NSAIDs. This might be the reason why for PPIs side-effects on muscle function are described while effects on muscle wasting are not. There is, however, a knowledge gap for situations in which PPIs are provided in the absence of NSAIDs and for the situation in which the combination treatment of PPIs with NSAIDs is stopped and gut microbiota might be altered, as described in the next paragraph.

### 3.2. Alterations in Gut Microbiota Can Contribute to Both Muscle Wasting and Obesity

An increase in *Enterobacteriaceae* can be seen in both cachectic patients and in PPI-users, which results in increased LPS production and activation of TLR4, increasing inflammatory status [9]. Moreover, animal data indicate that the PPI omeprazole reduces microbiota diversity. In [9], microbiota-derived formate levels were increased. Microbiota-derived formate levels have been associated with increased intestinal inflammation. Next to that, the authors showed an association between a low dietary magnesium bioavailability and formate levels. These data indicate that changes in microbiota might occur due to PPI use [82]. Next to that, increased TLR4 stimulation by LPS has been reported to increase hypothalamic inflammation, inducing disease-induced anorexia and loss of muscle mass and function [83,84]. Bacteria in the gut also produce metabolites and contain structural components that act as signaling molecules to enteroendocrine cells in the mucosa. These cells in turn produce regulatory hormones (e.g., CCK, PYY, GLP-1, and serotonin) in key metabolic processes such as appetite regulation, glucose tolerance, and fat storage in the body [85,86]. Alterations in the gut microbiota can, therefore, impact metabolic disorders such as cachexia, either directly, via metabolic deregulation, or indirectly, by contributing to the underlying inflammatory processes of chronic illness. The impact of gut health on cachexia has recently been shown in animal models for cancer cachexia [20]. In COPD patients, there are indications of decreased splanchnic perfusion and an impaired barrier function, leading to a reduction in the uptake of nutrients from the gut and contributing to inflammation [87]. Moreover, alterations in microbiota also play a key role in the development of obesity. There are many mechanisms involved, such as increased energy uptake from the food due to fermentation of the gut microbiota and influencing appetite regulation and metabolism via signaling molecules [88,89]. 

## 4. PPI Use and Muscle Wasting in the Presence of Obesity

Obesity is associated with magnesium and vitamin D deficiencies; metabolic disturbances, such as insulin resistance; and chronic low-grade inflammation. Given the key role for inflammation in the development of muscle wasting, PPI use might further worsen the condition of an obese patient by increasing muscle wasting via an increase in inflammatory responses.

### 4.1. Low Magnesium Levels Are Often Seen in Obesity

Magnesium deficiencies have been reported frequently in obesity [90,91,92,93,94,95,96,97,98,99,100,101]. Different causal factors between obesity and hypomagnesemia have been suggested. A relationship between insulin sensitivity and magnesium levels [94,98,102,103] and an inadequate intake due to a poor quality of the diet [104] were reported as key factors contributing to the association between obesity and low magnesium levels. This poor nutritional intake can further worsen in the presence of a restricted diet, if this is not supported by nutritional counseling. The dietary guidance for patients with heart failure (HF) [105], for example, states that next to the traditional focus on sodium and fluid intake restriction, adequate intake of macro- and micronutrients should be monitored and properly counseled as well, because it may influence morbidity and mortality [105]. The link with insulin sensitivity rather than with obesity further supports the need to look into obese phenotypes, since the visceral obese inflamed phenotype is also related to the presence of insulin resistance. Another important causal factor for low magnesium levels in obesity is the increased blood levels of non-esterified or free fatty acids that directly bind to magnesium and so reduce the free magnesium concentration in the blood [106].

### 4.2. Low Magnesium Levels Lead to Insulin Resistance

In obesity, a chronic, low-grade inflammatory state is involved in the development of insulin resistance (IR), metabolic syndrome, and type II diabetes mellitus (T2DM) [107]. Currently, more awareness is also rising of the fact that low magnesium levels occur at higher frequency in diabetic patients [98]. The underlying mechanism has been elucidated to a certain extent. Insulin stimulates magnesium uptake in insulin-sensitive tissues like muscle. Subsequently, the magnesium is used by the tissue to form magnesium-ATP complexes. Lower levels of magnesium-ATP complexes result in insulin resistance, reducing muscle glucose utilization. For the muscle, this implies lower energy status, resulting in increased muscle breakdown. Next to that, magnesium is involved in insulin signaling. Lower intracellular pancreatic Mg levels result in a reduction of insulin secretion [98]. 

### 4.3. Vitamin D Deficiency Is Prevalent in Obesity

Systematic reviews and meta-analyses have shown that obese individuals are at a greater risk for vitamin D deficiency [108,109], although the intake of vitamin D from food or supplements is not reduced in obese individuals [109]. This can be explained by the finding that researchers showed an inverse association between BMI and the response to vitamin D supplementation to increase serum vitamin D levels. It is hypothesized that the increased amount of adipose tissue withdraws more fat-soluble vitamin D in obese individuals compared to non-obese individuals, leading to a decreased bioavailability of vitamin D from cutaneous and dietary sources [110]. Vitamin D deficiencies should be avoided, because adequate vitamin D levels in obese individuals are correlated with an increase in adiponectin. This adipocytokine is involved in regulation of glucose levels and fatty acid breakdown and can so help reduce the metabolic disturbance and the development of insulin resistance as seen in obesity [111].

Moreover, adequate levels of vitamin D act as a suppressor of pro-inflammatory cytokines IL-6 and TNFα [112]. These cytokines are upregulated in the obese state and contribute to metabolic disturbance [113]. A deficiency of vitamin D in obesity might contribute to the increased inflammatory state that is already seen in obesity and that contributes to sarcopenic obesity. 

### 4.4. PPI Use Might Increase Prevalence of Micronutrient Deficiences and Enhance Development of Sarcopenic Obesity

**Figure 2:**
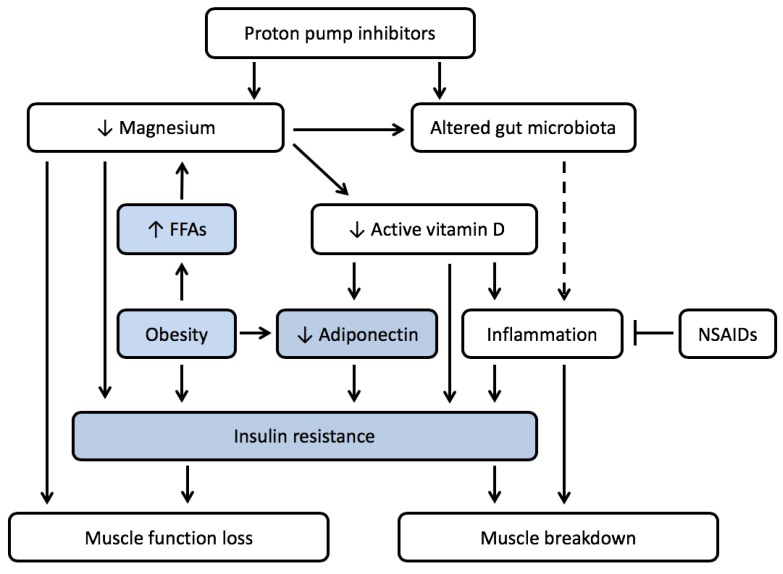
The proposed mechanism by which the use of proton pump inhibitors can lead to increased muscle breakdown in the presence of obesity. The use of proton pump inhibitors may lead to an increase in chronic low-grade inflammation by altering the gut microbiota and by lowering magnesium and vitamin D levels. Obesity contributes to the presence of low magnesium levels via increased free fatty acids (FFAs) and insulin resistance (directly and via lower adiponectin levels). The increase in inflammation also contributes to insulin resistance. Low magnesium levels and insulin resistance both lead to muscle function loss. The increased inflammation, together with insulin resistance, lead to muscle breakdown. This leads to the development of sarcopenic obesity. When PPIs are given together with nonsteroidal anti-inflammatory drugs (NSAIDs), the (increase in) inflammation is likely diminished, leading to no additional PPI-induced muscle breakdown. The impact of PPI use on muscle function is likely not strongly affected by NSAIDs, and might, therefore, occur both in the presence and absence of PPIs. The obesity-related mechanisms that contribute to muscle function loss and muscle breakdown are highlighted in blue.

The presence of obesity might aggravate an effect of PPIs on muscle wasting and lead to the development of sarcopenic obesity. In obesity, the development of insulin resistance and lower levels of magnesium, vitamin D, and adiponectin contribute to chronic low-grade inflammation, the underlying factor for muscle wasting. Adequate levels of both vitamin D and magnesium might prevent this effect and contribute to a decrease of the inflammatory state and a reduction in metabolic disturbances, such as insulin resistance. Because the use of proton pump inhibitors is likely to increase the severity of these deficiencies, the use of proton pump inhibitors might contribute to an enhancement of the mechanisms involved in the development of sarcopenic obesity (Figure 2). 

## 5. Discussion

In this paper, we hypothesized that long term use of proton pump inhibitors contributes to an increase in symptoms of cachexia and sarcopenic obesity in patients with chronic illness. This effect might be mediated by negatively influencing the status of magnesium and by alterations in the gut microbiota. Magnesium deficiency in turn, might negatively impact the level of active vitamin D. A low magnesium status, lower levels of active vitamin D, and a less healthy gut microbiota might all contribute to the state of chronic low-grade inflammation in chronic illness that is a key symptom in the development of muscle wasting in the presence or absence of obesity. Next to that, a less healthy microbiota might also directly contribute to the metabolic deregulation in cachexia and sarcopenic obesity.

Figure 1 summarizes the hypothesized pathway from PPI-induced changes in magnesium and the microbiota to loss of muscle function in chronic illness-induced (pre-)cachexia. Concerning the PPI-induced changes, the change in pH induces changes in gut microbiota and receptor-mediated uptake of magnesium. Low magnesium levels lead to decreased muscle function. A reduced level of magnesium also reduces the activation of vitamin D, and alters the gut microbiota as well. Both an altered microbiota and reduced levels of magnesium and active vitamin D can contribute to the inflammation that is already present due to the chronic illness and the presence of (pre-)cachexia. In this way the inflammatory status worsens and may lead to increased muscle breakdown. NSAIDs are often prescribed in chronic illnesses and often combined with the use of PPIs to reduce side-effects. When PPIs are given together with NSAIDs, the (increase in) inflammation is likely to be reduced, leading to less muscle breakdown. The impact of PPI use on muscle function loss might, however, not be corrected by NSAIDs. Moreover, the effect on microbiota might remain after termination of the combined NSAID and PPI treatment. Whether PPI use has a negative effect on muscle function and muscle breakdown in the context of NSAID use, therefore, needs further investigation. 

Figure 2 summarizes the hypothesized pathway from PPIs-induced changes in the presence of obesity that may lead to the development of sarcopenic obesity. Next to the pathways discussed in Figure 1, in this case the obese state influences magnesium levels itself via an increase in free fatty acids, contributes to the development of insulin resistance via a decrease in vitamin D and adiponectin, and leads to the development of muscle wasting or sarcopenic obesity. It is likely that in the presence of visceral adipose tissue-induced inflammation, as is present in sarcopenic obesity, the inflammatory status can be further affected by use of PPIs, leading to accelerated muscle breakdown. This, however, needs further investigation. In view of these findings, possible side effects of PPIs in patients with chronic illnesses susceptible to the development of cachexia, including sarcopenic obesity, merit further study.

The question that remains is whether the benefits of PPI use outweigh the risks in chronically ill patients with risk of muscle mass and function loss. If properly prescribed [37], it is likely that they do. PPIs contribute extensively to reductions in all kinds of upper gastro-intestinal complications and their prevention if NSAIDs are taken [37,114]. Proton pump inhibitor use has also been reported to be associated with a lower risk of acute exacerbation and mortality in COPD patients with gastroesophageal reflux disease [115]. Moreover, the level of evidence of the risks of PPIs is still low. One should also take into account that the possibility of a relation of PPI with sarcopenia was based on a study with a limited number of subjects (*n* = 200). The paper, however, does not indicate if corrections for confounding factors were made. This sarcopenic population is older, has a higher prevalence of co-morbidities, and has poly-drug use, which might all contribute to confounding factors. On the other hand, are the data obtained with a data-driven machine-learning model to characterise factors involved, which are to a certain extend unbiased. Still, causal relationships need to be further investigated.

The mechanistic aspects described in this paper are in part based on animal research. In humans, the relationships between PPI use and magnesium levels or between magnesium and inflammation were investigated only in epidemiological studies or described in case reports [52,54,116]. For our hypothesis of PPI use leading to muscle function loss, most support from literature is available. For the effect of PPI on magnesium levels, a recent meta-analysis indicated a 1.4-fold increase on the risk of developing hypomagnesia in PPI-users [117]. However, authors indicate that heterogeneity in de data reduced the level of evidence, and therefore, more studies are needed to come to a high level conclusive answer. The in Figure 1 and Figure 2, predicted side effects of PPI use, however, might add to existing muscle function loss and might be prevented by nutritional interventions, which warrants further investigation.

The relationship between PPI use and muscle protein breakdown has not been described in the literature. In practice, PPIs are (most of the time) provided together with NSAIDs, which means that the possible inflammatory effect of PPIs might be superseded by the anti-inflammatory effect of the NSAIDs. There is, however, some data supporting a PPI-induced change in microflora. This indicates that after termination of the combined PPI and NSAID treatment, inflammation might be induced by the still-altered gut microbiota. It is, however, purely hypothetical because it is not possible to establish a direct cause and effect relationship based on the data available. 

Magnesium and vitamin D deficiencies and alterations in the gut microbiota have been reported in chronic diseases such as cancer, heart failure, and COPD. In cancer, multiple pathological changes contribute to an altered energy metabolism and anorexia, which both contribute to nutrient deficiencies [12]. Gut barrier dysfunction and bacterial translocations are associated with cancer, also in the absence of chemotherapy [20,118]. Patients with heart failure have a low intake of several micronutrients, such as magnesium, vitamin D, and iron. These micronutrients are important cofactors for normal cardiac metabolism and deficiencies are often seen during progression of the disease [119,120]. The development of anorexia and malnutrition further contributes to the development of micronutrient deficiencies in patients with advanced heart failure [14]. Moreover, alterations in the gut were reported in those patients: ischemia of the intestinal mucosa may lead to an increased gut permeability and bacterial translocation [121]. In COPD patients, micronutrient deficiencies are also common; in particular, for vitamin D, vitamin B12, and iron [122,123,124].

Another point to consider is that interpretation of the relationship between magnesium levels and health is difficult for two main reasons. First, discussions are ongoing on the current serum magnesium reference interval of 0.70–0.95 mmol/L [125]. Second, only 0.8% of the total magnesium in the body is available in serum [48], making it questionable whether serum magnesium is a sensitive and specific biomarker of magnesium status [126]. Serum magnesium levels can still be in the normal range, even when body stores of magnesium are depleted. In addition, the exact mechanisms by which PPI induce magnesium deficiency are still being investigated. It is, however, reported frequently that long term PPI use can decrease magnesium levels [35,36,37,38] and that low magnesium levels are associated with increased risk of chronic illness [47]. More insight into the impact of PPI use on magnesium status and a more reliable biomarker of magnesium status is necessary.

To summarize, patients with chronic illness have been described to be susceptible to develop micronutrient deficiencies and an altered gut microbiota. PPI use has been linked to both phenomena, but causal relationships need further investigations [37,127,128,129]. Therefore, we suggest monitoring the micronutrient status in patients on long term use of proton pump inhibitors. In case of a vitamin D deficiency, supplementation of vitamin D together with magnesium might be considered if magnesium intake or blood levels are low.

## 6. Conclusions

The mechanisms and relationships described in this review underline that use of proton pump inhibitors might add to the symptoms of muscle function loss in patients with chronic illness. There is a need for prospective studies among these patients to investigate whether PPI use increases muscle function loss and inflammation, during and after use of PPIs, with special attention for gut microbiota, and vitamin D and magnesium status. 

## Abbreviations

BMI	body mass index
CCK	Cholecystokinin
COPD	chronic obstructive pulmonary disease
FFAs	free fatty acids
GLP-1	Glucagon-like peptide 1
HF	heart failure
IFN-γ	interferon-γ
IL-1	interleukin-1
IL-6	interleukin-6
IR	insulin resistance
MHOB	Metabolically Healthy Obesity
MONW	Metabolically Obese Normal Weight
MUO	Metabolically Unhealthy Obesity
NFκB	Nuclear Factor kappa B
NMDA	*N*-methyl-d-aspartate
NSAIDs	nonsteroidal anti-inflammatory drugs
PGE2	prostaglandin E2
PPIs	proton pump inhibitors
PYY	Peptide YY
T2DM	type II diabetes mellitus
TNF-α	tumor necrosis factor- α
TRPM6	transient receptor potential melastatin 6
TRPM7	transient receptor potential melastatin 7
VDBP	vitamin D binding protein
